# Leading by Example: Identity Leadership and Mental Health in Men’s Sheds Members

**DOI:** 10.1177/07334648241289020

**Published:** 2024-09-30

**Authors:** James J. Clarke, S. Alexander Haslam, Leah S. Sharman, Briana Guerrini, Kirsten Holmes, Rebecca Talbot, James Wild, Peter M. McEvoy

**Affiliations:** 1 1649 Curtin School of Population Health, Faculty of Health Sciences, Curtin University, Perth, Western Australia, Australia; 2 1974 Curtin enAble Institute, Faculty of Health Sciences, Curtin University, Perth, Western Australia, Australia; 3 School of Psychology, Faculty of Health and Behavioural Sciences, The University of Queensland, Brisbane, Queensland, Australia; 4 School of Management and Marketing, Curtin University, Perth, Western Australia, Australia; 5 Men’s Sheds of Western Australia, Perth, Western Australia, Australia; 6 Centre for Clinical Interventions, North Metropolitan Health Service, Perth, Western Australia, Australia

**Keywords:** Men’s sheds, mental health, identity leadership, path analysis

## Abstract

The impact of leadership on members’ mental health outcomes in community-based mutual-aid organizations such as Men’s Shed is unknown. We analyzed (a) whether identity leadership is associated with Shed members’ mental health, and (b) whether these links are mediated by psychological safety, social network quality, and social identity. Path analysis on data collected from 162 Australian Men’s Shed members revealed statistically significant associations between identity leadership and each mechanism, and our model accounted for significant variance in mental health outcomes (14%–24%, *p*s < .001). Only social network quality and psychological safety were associated with unique variance in mental health outcomes. All indirect effects via social network quality and psychological safety were significant. These findings suggest the proposed mechanisms explain the relationship between identity leadership mental health outcomes in mutual-aid organizations such as Men’s Sheds.

**What this paper adds**

• Identity leadership may indirectly affect psychological wellbeing in older men who participate in community-based mutual-aid groups such as Men’s Sheds.
• Improved social network quality within one’s Shed appeared to be a key mechanism that may explain such relationships.

**Applications of study findings**

• Leaders of community mutual-aid organizations aiming to improve wellbeing in older men may increase their effectiveness through embodying identity leadership principles.
• Efforts to promote social network quality within one’s Shed may also be a significant pathway to improve psychological wellbeing in community-based mutual-aid groups.

## Introduction

Men’s Sheds are community-based mutual-aid organizations that engage members in various hobbies while fostering social connection in a non-threatening environment. Men’s Sheds membership has been associated with positive impacts on health and wellbeing (Foettinger et al., 2022; McGrath, Murphy, & Richardson, 2022). Although some possible explanatory pathways for these observations have been drawn from qualitative studies (Kelly et al., 2019), there have been few quantitative examinations of these pathways. Elsewhere, though, wellbeing outcomes in group members have been linked to *leadership*—particularly in organizational and occupational contexts (Montano et al., 2023). The relationship between leadership and member wellbeing may also be of particular importance for community-based organizations such as Men’s Sheds with objectives to improve the wellbeing of members. However, precisely what forms of leadership might contribute to positive health outcomes in Men’s Sheds or other similar community contexts has yet to be clarified.

### How do Community Organizations Positively Impact on Wellbeing?

Similar to the goals of social health initiatives such as social prescribing (Dingle & Sharman, 2022), activities in Men’s Sheds are intended to be broadly appealing, particularly for people who experience barriers to traditional health initiatives (Foettinger et al., 2022; McGrath, Murphy, Egan, et al., 2022). Kelly and colleagues’ (2019) model identifies possible mental health benefits of Men’s Shed membership and highlights factors that mediate these outcomes. Key mental health benefits identified by their model include lower rates of depression and loneliness, higher rates of meaning in life and general wellbeing, and increased social connection, through multiple mediating pathways. However, their analysis is informed primarily by qualitative observations and, although these provide important insights and solidarity to the theoretical framework, the robustness and generalizability of their conclusions are open to question. McEvoy and colleagues (2023) extended this model by finding quantitative empirical support for Men’s Shed engagement predicting wellbeing, loneliness, meaning in life, and social networks within Shed members located in Western Australia. Clarke and colleagues (2023) provided further quantitative evidence for Shed factors promoting positive psychological wellbeing in members located in Western Australia. However, there have been relatively few quantitative investigations of the health benefits of Men’s Sheds (e.g., Clarke et al., 2023; McEvoy et al., 2023; McGrath, Murphy, & Richardson, 2022), and even fewer studies of the processual pathways between Shed membership and mental health outcomes.

### Leadership as a Driver of Wellbeing Outcomes

Reviews and meta-analyses consistently observe associations between leadership and mental health in occupational contexts (Montano et al., 2017, 2023). Destructive leadership is generally found to contribute to poor mental health among organizational members, whereas transformational, relationship-oriented, and task-oriented leadership generally contribute to positive mental health (see Montano et al., 2023). However, the generalizability of these findings to the Men’s Shed and other similar community contexts is potentially compromised by the fact that they relate to occupational contexts in which the context of management and leadership is qualitatively different. For example, Men’s Shed members do not receive financial remuneration and their motivations to attend are likely to be very different to those of employees in an occupational context. Leadership in these community contexts can be volunteer-led with greater hierarchical fluidity and interpersonal closeness between members (Gammage & Foster, 2017). Moreover, previous findings offer limited insight into what leaders *actually need to do* to promote good health, other than to focus on relationships and tasks and to not be destructive. Relationships and tasks are undoubtedly important, but which relationships and which tasks should a leader prioritize?

We know that leadership can positively affect group members’ mental health through its capacity to increase psychological safety, social network quality, and social identification (e.g., Fransen et al., 2020; Haslam & Reicher, 2016; O’Donovan & McAuliffe, 2020). Each of these constructs are also associated with positive mental health outcomes (e.g., Bui, 2020; Chui, 2018; Domènech-Abella et al., 2021). More specifically, psychological safety is thought to promote wellbeing by affording group members opportunities to participate, develop, and perform more effectively (Edmonson & Lei, 2014). On the other hand, social network quality is thought to promote wellbeing because it helps to satisfy group members’ basic emotional needs while mitigating the negative consequences of social isolation and loneliness (Suragarn et al., 2021). Social identification also provides a basis for people’s sense of psychological connection to a group (Haslam et al., 2022), and this is hypothesized to unlock a range of social and psychological resources that support mental health; notably social support and a sense of meaning, purpose, agency and collective self-efficacy (Cruwys et al., 2014; Haslam et al., 2018), with some support existing in the Men’s Shed literature (Ford et al., 2015).

In the Men’s Shed context, this emphasis on the importance of psychological safety, social network quality, and social identification is consistent with Kelly and colleagues’ (2019) model, which posits that improved social connections with other members, belongingness, and a sense of safety and security are interconnected pathways through which Shed membership can improve mental health. Accordingly, we propose that leadership that helps to promote these factors has the capacity to increase Shed members’ wellbeing and sense of meaning in life, while simultaneously reducing loneliness and depression.

### What Form of Leadership Might Best Have an Impact?

In contrast to traditional approaches to leadership that focus on the importance of leaders’ personal qualities and style, the social identity approach argues that leadership is grounded in the sense of shared identity between leaders and group members that leaders play a central role in developing (Haslam & Reicher, 2016). In particular, this approach suggests that leaders do this through *identity leadership* that has four key components: (1) *identity entrepreneurship* in which they craft a sense of shared identity with and for the group so as to create “a sense of us,” (2) *identity prototypicality* in which they embody that sense of shared social identity and are thereby seen as “one of us,” (3) *identity advancement* in which they promote the interests of shared identity and so are seen to be “doing it for us,” and (4) *identity impresarioship* in which they devise structures and activities that embed a sense of shared social identity and thereby “make us matter” (Haslam et al., 2011). Leadership, then, may serve to create, represent, and advance a sense of shared social identity within a given group (i.e., a sense of “us-ness”; Haslam et al., 2017).

The social identity approach to leadership therefore sees social and psychological connectedness as integral to the practice of leadership (Haslam & Reicher, 2016). This emphasis also aligns closely with motivations that Shed members themselves identify as central to both their participation and their wellbeing (Foettinger et al., 2022; Ford et al., 2015). Though the value of identity leadership is gaining more popularity in the literature, to date, there has not been an investigation of identity leadership and mental health outcomes in older adult contexts. Thus, conceptualizing leadership through a social identity prism could provide a useful framework for understanding the links between leadership and health in older adults and in Men’s Shed contexts and other activity-based community groups.

### The Current Study

**Figure 1.**
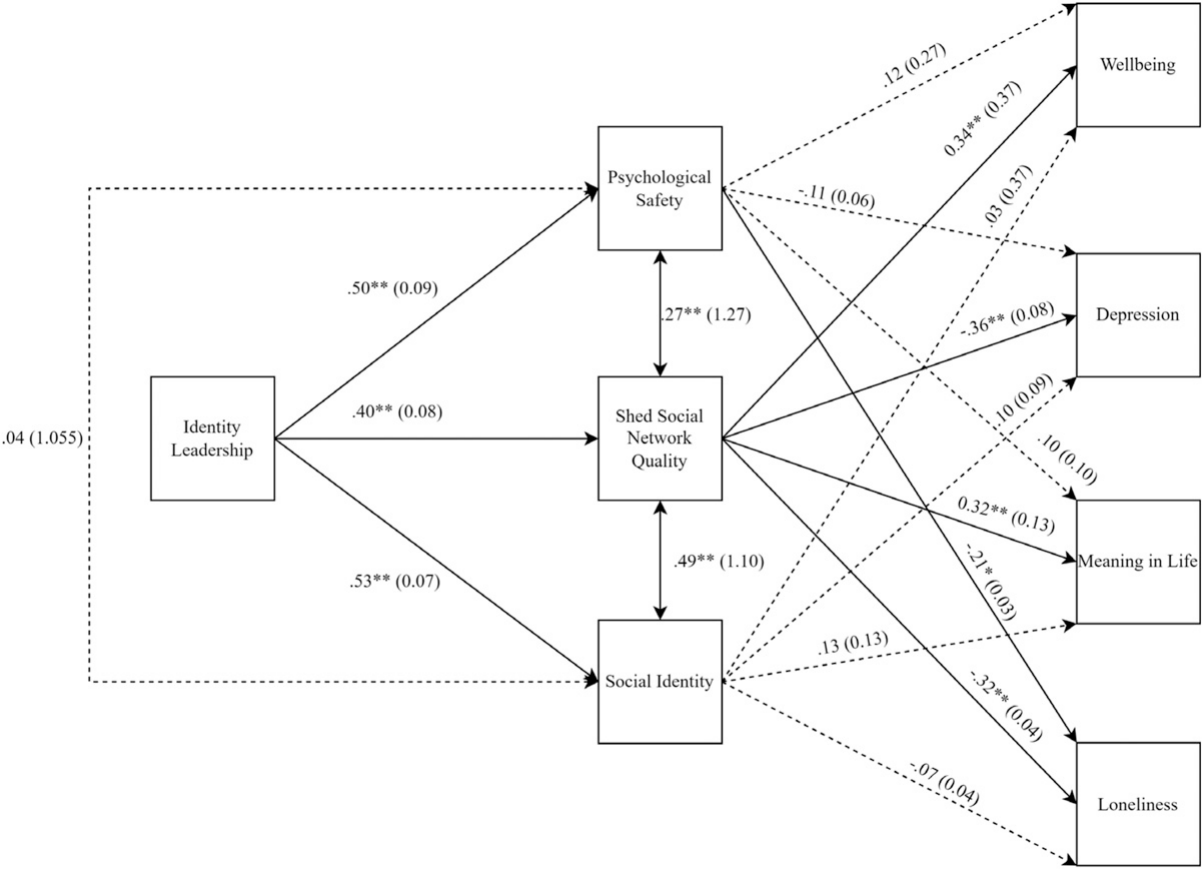
Path analysis model.

The present research sought to explore the relationship between identity leadership and members’ mental health across a network of Western Australian Men’s Sheds. Wellbeing, depression, meaning in life, and loneliness have all previously been identified as aspects of mental health that are linked to membership of Men’s Sheds (Kelly et al., 2019; McEvoy et al., 2023), and consequently we included these as key indicators of mental health. We hypothesized that identity leadership would be associated with mental health outcomes indirectly through psychological safety, social network quality, and social identity. Due to the close theoretical links between psychological safety, social network quality, and social identification (i.e., as aspects of a strong sense of shared social identity; e.g., Fransen et al., 2020; McEvoy et al., 2023), we also expected these variables to covary (see Figure 1).

## Method

### Design, Participants, and Procedure

We adopted a cross-sectional exploratory design for our study using data gathered from an online survey of Men’s Shed members using Qualtrics software. Ethical approval was obtained from the Curtin University Human Research Ethics Committee [HRE2022-0148]. Participants completed information and consent forms before completing the survey. Each measure was created as a separate block on Qualtrics and the order in which the measures were presented to participants were randomized. The data were collected at the second timepoint of a three-wave longitudinal study. This second wave of data collection occurred between November 20, 2022 and January 31, 2023. Participants were initially recruited through advertisements at their local Sheds and were offered the chance of winning one of ten $50 hardware store vouchers for participation.

Data were collected from 179 potential participants from 52 Men’s Sheds from a total population of over 180 Men’s Sheds in Western Australia. Seventeen cases were removed due to duplicate responses, not meeting inclusion criteria, providing incomplete data, or not confirming consent, resulting in a final sample size of 162 participants. Four participants provided incomplete data on at least one variable in our model. All participants were male and between 22.94 and 91.67 years of age (*M* = 71.90, *SD* = 9.03; *N* = 10 < 60 years, 1 participant did not disclose age). Participants had been members of their Shed for an average of 5.56 years (SD = 4.64). Most were retired, attended urban Sheds, and attended at least fortnightly. Members had varying educational backgrounds with a university degree being the most common level of educational attainment (see Table 1 for more details).

**Table 1.** Participant Demographics.

Variable	*N* = 162
Employment status	
Retired	135 (83.33%)
Working	24 (14.82%)
Unemployed	3 (1.85%)
Shed location	*n* = 159
Urban	115 (70.99%)
Rural	41 (25.31%)
Split	3 (1.85%)
Frequency of Shed attendance	*n* = 161
Weekly	136 (87.47%)
Fortnightly	11 (6.83%)
Monthly or less	14 (8.69)%
Education level	*n* = 161
University degree	75 (46.58%)
High school certificate	32 (19.88%)
Diploma	30 (18.63%)
Tertiary certificate	24 (14.91%)

### Measures

#### Identity Leadership

The Identity Leadership Inventory - Short Form (ILI-SF; Steffens et al., 2014) measures the extent to which group leaders embody and promote social identity, with four items that capture one of the four dimensions of identity leadership (entrepreneurship: “*the leaders of my Men’s Shed create a sense of cohesion within the group*”; prototypicality: “*the leaders of my Men’s Shed are model members of the group*”; advancement, “*the leaders of my Men’s Shed are champions of the group*”; and impresarioship, “*the leaders of my Men’s Shed create structures that are useful for the group*”). Participants responded to these statements on a 5-point Likert scale (where 1 = *strongly disagree* and 5 = *strongly agree*). Scores range between 4 and 20, with higher scores indicative of more effective identity leadership. The ILI-SF has demonstrated good psychometric properties (Steffens et al., 2014) and high internal consistency our sample (α = .83).

#### Shed Psychological Safety

The Team Psychological Safety Scale (TPSS; Edmondson, 1999) comprises seven items such as “*It is safe to take a risk on this team*” on a 5-point Likert scale ranging from *strongly disagree* (1) to *strongly agree* (5). Total scores could range between 7 and 35 with higher scores indicating a greater degree of perceived psychological safety. The TPSS has demonstrated acceptable psychometric properties in previous research (e.g., Fransen et al., 2020) and adequate internal consistency our sample (α = .75).

#### Shed Social Network Quality

Three items from the Lubben Social Network Scale - 6 item version (LSNS-6; Lubben et al., 2006) assessed social network quality within one’s Shed. The LSNS-6 is comprised of two subscales that assess social network quality in reference to family and friends. As we were interested in the Men’s Shed context, items were framed specifically with reference to this (e.g., “*How many Men’s Shed members do you see or hear from at least once a month?*” and “*How many Men’s Shed members do you feel at ease with that you can talk about private matters?*”). Responses were made on 6-point Likert scales ranging from *none* (0) to *nine or more* (5). Scores could range between 0 and 18 with higher scores indicating greater Shed social network quality. The LSNS has demonstrated good psychometric properties (Gray et al., 2014) and had high internal consistency in our sample (α = .86).

#### Social Identification

A three-item version of the Single-Item Social Identification Scale (SISI; Postmes et al., 2013) assessed social identity, and included items such as “*I identify with my local Men’s Shed*” on a 5-point Likert scale ranging from *strongly disagree* (1), to *strongly agree* (5). Scores could range between 3 and 15 with higher scores indicating greater identification. This measure has demonstrated good psychometric properties (Postmes et al., 2013) and had high internal consistency in our sample (α = .79).

#### Wellbeing

The World Health Organization-Five Wellbeing Index (WHO-5 Index; WHO, 1998) assessed general wellbeing. Items such as “*I have felt cheerful and in good spirits*” were rated on 6-point Likert scales ranging from *at no time* (0) to *all of the time* (5). Total scores range between 0 and 25 with higher scores indicating greater wellbeing. For ease of interpretation, these scores are multiplied by 4 to provide a total score out of 100. The WHO-5 Index has good psychometric properties (Bonnín et al., 2018) and had high internal consistency in our sample (α = .88).

#### Depression

Depression was assessed using the Patient Health Questionnaire - 9 item version (PHQ-9; Kroenke et al., 2001), which asks respondents to consider how much they have been bothered by a specific symptom over the past two weeks (e.g., “feeling down, depressed, or hopeless”). Responses were made on 4-point Likert scales ranging from 0 *(not at all*) to 3 (*nearly every day*). Scores range between 0 and 27 with higher scores indicating more depressive symptoms. The PHQ-9 has adequate psychometric properties (Titov et al., 2011) and had high internal consistency in our sample (α = .84).

#### Meaning in Life

Meaning in life was assessed using the “Presence” subscale of the Meaning in Life Questionnaire (MLQ; Steger et al., 2006), which assesses the extent to which an individual has a sense of meaning in life. The Presence subscale comprises five items (e.g., “*I understand my life’s meaning*”) rated on 7-point Likert scale ranging from *absolutely untrue* (1) to *absolutely true* (7). Scores range between 1 and 35 with higher scores indicating a greater presence of meaning in life. The MLQ has adequate psychometric properties (Rose et al., 2017) and had high internal consistency in our sample (α = .89).

#### Loneliness

The University of California Los Angeles Loneliness Scale - 4 item version (UCLA-4; Russell et al., 1980) measures loneliness with four items (e.g., “*no one really knows me well*”) on a 4-point Likert scale that ranges from *never* (1) to *always* (4). Scores range between 4 and 16 with higher scores indicating greater degrees of loneliness. The UCLA-4 has good psychometric properties (Alsubheen et al., 2023) and had adequate but lower than ideal internal consistency in our sample (α = .65).

### Analysis

We used the pathj—Path Analysis 0.9.0 package in the jamovi v. 2.3.28 software to analyze our hypothesized indirect effects model (see Figure 1). Identity leadership was the exogenous variable and all remaining variables were endogenous. Psychological safety, social network quality, and social identification were intermediate variables between identity leadership and health outcomes. Covariances were freed between the intermediate variables (see Figure 1). A Monte Carlo simulation with 162 observations, 1000 replications, direct effects of predictors of .30, covariances of .30, and indirect effects of .09 on wellbeing, depression, meaning in life, and loneliness revealed at least 93% power to detect effects and at least 93% coverage (i.e., 93% of the simulated 95% confidence intervals contained the true effect). Therefore, the sample size was deemed sufficient to detect theorized relationships between variables. The models were re-run restricting the participants to individuals aged 60 years and older, while controlling for age, employment status, and education level. As the pattern of significant findings was identical, only the results from the initial analyses are reported.

## Results

There were significant zero-order correlations for all variables in expected directions, except for non-significant correlations between depression and both identity leadership and social identity (see Supplemental Materials). The hypothesized model explained a significant proportion of variance in wellbeing (18%, *r*^2^ = .18, *p* < .001), depression (14%, *r*^2^ = .14, *p* < .001), meaning in life (21%, *r*^2^ = .21, *p* < .001), loneliness (24%, *r*^2^ = .24, *p* < .001), psychological safety (25%, *r*^2^ = .25, *p* < .001), social network quality (16%, *r*^2^ = .16, *p* < .001), and social identification (19%, *r*^2^ = .19, *p* < .001).

As shown in Figure 1 and Table 2, identity leadership was positively associated with psychological safety, social network quality, and social identity. Social network quality significantly covaried with psychological safety and social identity, however, psychological safety and social identity did not. Psychological safety was associated with lower loneliness, but not with wellbeing, meaning in life, or depression. Social network quality was associated with greater wellbeing and meaning in life, and with lower depression and loneliness. Social identification did not predict wellbeing, meaning in life, depression, or loneliness.

**Table 2.** Parameter Estimates and Covariances.

Dependent	Predictor	β	Estimate	SE	95% Confidence Intervals	
					Lower	Upper
Wellbeing	PS	.12	.42	.27	−.12	.95
	SSNQ	.34**	1.36**	.37	.64	2.07
	SI	.03	.14	.37	−.59	.87
Dep	PS	−.11	−.09	.06	−.21	.03
	SSNQ	−.36**	−.32**	.08	−.49	−.16
	SI	.10	.09	.09	−.07	.26
MIL	PS	.10	.13	.10	−.06	.32
	SSNQ	.32**	.46**	.13	.21	.72
	SI	.13	.19	.13	−.07	.46
Loneliness	PS	−.21*	−.09*	.03	−.15	−.02
	SSNQ	−.32**	−.15**	.04	−.23	−.07
	SI	−.07	−.04	.04	−.12	.05
PS	IL	.50**	.67**	.09	.49	.85
	SSNQ ^a^	.27**	4.23**	1.27	1.74	6.71
SSNQ	IL	.40**	.48	.08	.31	.64
	SI ^a^	.49**	6.11**	1.09	3.96	8.26
SI	IL	.53**	.58**	.07	.44	.72
	PS ^a^	.04	.53	1.05	−1.54	2.59

*Note.* IL = identity leadership, SI = Social identity, PS = psychological safety, SSNQ = Shed Social Network Quality, Dep = depression, MIL = meaning in life, ^a^ covariance, * *p* < .01, ** *p* < .001.

The indirect effects of identity leadership through Shed social network quality significantly predicted wellbeing (B = .65, *β* = .14, *SE* = 0.21, *p* < .01, 95% CI = 0.24, 1.06), depression (B = −.15, *β* = −.15, *SE* = 0.05, *p* < .01, 95% CI = −.25, −.06), meaning in life (B = .22, *β* = .13, *SE* = 0.07, *p* < .01, 95% CI = .08, .36), and loneliness (B = −.07, *β* = −.13, *SE* = 0.02, *p* < .01, 95% CI = −.12, −.02). The indirect effect of identity leadership on loneliness through psychological safety was also significant (B = −.06, *β* = −.10, *SE* = 0.02, *p* < .01, 95% CI = −.10, −.01). All other indirect effects of identity leadership on outcomes through psychological safety and social identity were non-significant (*p*s > .05).

## Discussion

We explored how leadership within a Men’s Shed may contribute to members’ mental health outcomes. As hypothesized, identity leadership was associated with wellbeing, depression, meaning in life, and loneliness indirectly through mechanisms of psychological safety and social network quality. Psychological safety and social identity significantly covaried, and they both significantly covaried with Shed social network quality. Furthermore, Shed social network significantly predicted unique variance in all outcome variables, and psychological safety significantly predicted unique variance in loneliness. However, not all hypothesized pathways were supported. Psychological safety did not uniquely predict wellbeing, depression, or meaning in life, and social identity did not significantly predict unique variance in any outcome. Overall, our pattern of results suggests that identity leadership is associated with Shed member mental health outcomes predominantly through higher Shed social network quality, and that this relationship may be strengthened by the direct relationships between identity leadership and psychological safety and social identity. Our study is the first to attempt to explore these relationships in the context of a mutual-aid organization such as Men’s Sheds. Additionally, as our sample was comprised mostly of older men (*M* = 71.90, *SD* = 9.03), our findings suggest that improving social connectedness and psychological wellbeing through Men’s Shed engagement may be of particular benefit for older men who experience barriers to engage with health initiatives (Galdas et al., 2005).

Our observation that identity leadership was associated with social identity, psychological safety, and social network quality supports and extends previous literature. Our findings provide empirical support for theory suggesting that identity leadership cultivates group social identity (Haslam & Reicher, 2016). It is possible that cultivating strong social identity through identity leadership reduces perceived costs to expressing contrary opinions or seeking help, consequently raising a group’s psychological safety (Fransen et al., 2020). Our findings replicate and extend previous literature by observing this relationship in the context of the Men’s Sheds organization and provide the first evidence for an association between identity leadership and social network quality. Further research is required to clarify what specific qualities members perceive to comprise identity leadership in the Men’s Shed context, but our findings suggest that Shed leaders embodying the prototypical qualities of Shed membership may assist to cultivate greater social identity, psychological safety, and social network quality for Shed members.

The pattern of covariances between psychological safety, social identity, and social network quality identifies a novel relationship as well as complements and contrasts findings observed in other contexts. Social identity may foster closer relationships and provide greater opportunities to make and maintain social connections, which may explain the significant relationship between these variables in our model. To the best of our knowledge, no such finding has been made in similar contexts previously. Psychological safety may promote social network quality by providing a fertile context for relationship development (McEvoy et al., 2023). Contrasting Fransen and colleagues’ (2020) findings, we did not observe a significant covariance between psychological safety and social identity. As psychological safety is often a consequence of leadership (Newman et al., 2017) and social identity is a group-level construct, it may be possible that one’s social identity is not sufficient to influence group psychological safety.

Finding that Shed social network quality predicted unique variance in all outcome variables suggests it may be a key mechanism through which identity leadership influences broad member outcomes, whereas psychological safety only uniquely predicted loneliness. Psychologically safe environments promote the perception that one is free to express vulnerability through asking for help or making mistakes (Edmondson, 1999), which may promote a perception of stronger connectedness, understanding, and acceptance from fellow group members, and thereby reduce perceived loneliness. Social network quality has previously been associated with wellbeing, depression, meaning in life, and loneliness in the Men’s Shed and other contexts (Bui, 2020; Chui, 2018; McEvoy et al., 2023). However, our study is the first to find identity leadership associated with these outcomes indirectly through Shed social network quality. Together with the observed covariances with psychological safety and social identity, this suggests that identity leadership may help establish stronger social networks within a Shed directly, and indirectly through psychological safety and social identity, which in turn may influence member mental health.

### Practical Implications

Our findings suggest that improving identity leadership in leaders of mutual-aid organizations such as Men’s Sheds may cultivate psychological safety, social network quality, social identity, and consequent improvements in mental health. For example, the 5R identity leadership program has emerging evidence to support its capacity to improve identity leadership knowledge and positive team/group outcomes (Haslam et al., 2023; Mertens et al., 2021). Our analysis was based on data from men who were already engaged in their Sheds and as such our findings suggest the importance of identity leadership to encourage greater participation and engagement, and subsequent mental health benefits once men become members of the organization. Therefore, Men’s Sheds and other mutual-aid organizations may more effectively promote member wellbeing through training Shed/group leaders to implement stronger identity leadership.

Social network quality being a potential key variable in contributing to Shed member outcomes suggests that interventions aimed at promoting improved relationships and connectedness among older men may also have a significant impact on mental health outcomes such as wellbeing, depression, meaning in life, and loneliness. Promoting such social engagement may be of particular importance for older men for whom social network size protects against risk of depression (Domènech-Abella et al., 2021). Although our findings suggest identity leadership may have a role in promoting this, there may be other means to promote social network quality such as ensuring that there are adequate opportunities for members to socialize and forge these relationships (e.g., structured break times to converse and organizing alternative social events for members).

As our findings further supports the benefits of Men’s Sheds for older men, considering means to encourage Shed participation appears to be a productive pursuit for optimizing positive mental health outcomes. As individual factors (e.g., pro-social attitudes, desire to keep busy, and significant life events) and social networks outside the Shed (i.e., female family members) appear to encourage Shed participation for some individuals (Flood & Blair, 2013; Milligan et al., 2015; Nurmi et al., 2018), broadening the points of contact with older men about Men’s Sheds may be a fruitful pursuit to attract individuals most at risk of mental ill-health. For example, health-care service providers (i.e., general practitioners) encouraging involvement, advertisement in the neighborhood (i.e., local newspapers), and a direct invitation from the local Men’s Shed champion may be productive in attracting new members (Nurmi et al., 2018).

### Future Directions

Numerous potential future research directions may prove fruitful in understanding how identity leadership may improve Shed member wellbeing. First, our proposed model may benefit from replication and expansion. Longitudinal replication of our model would provide higher quality evidence for the theorized causal and reciprocal relationships. Second, although we found that identity leadership is a potential contributor to mental health, the specific characteristics of what constitutes a prototypical social identity within the Men’s Shed context remains unexplored. Future research could elucidate what specific qualities and traits Men’s Shed members may believe best represents this social identity. Third, future research aimed at enhancing the benefits of Men’s Shed membership may benefit from targeting Shed social network quality, which had the strongest relationship with mental health outcomes in our model. Future research could extend this by trialling interventions that seek to improve Shed social network quality and observe impacts on mental health outcomes.

### Limitations

The limitations of our study should be considered while examining our findings and conclusions. First, our cross-sectional data precludes causal conclusions so research examining these relationships longitudinally is needed. Second, our sample was highly engaged in their Shed and consequently the proposed relationships between identity leadership and mental health outcomes may differ for newer Shed members or those who are not as highly engaged with the organization. Third, our sample was entirely comprised of members located in Western Australia. Therefore, findings may not generalize across different cultural contexts.

## Conclusion

Men’s Shed membership is associated with positive mental health outcomes. Although leadership within teams has been shown to link to mental health outcomes in other contexts, this relationship has not been previously explored in the context of mutual-aid organizations such as Men’s Sheds. For the first time, we found evidence that creating a sense of “us-ness” through identity leadership (where leaders cultivate, embody, and advance a sense of shared social identity within a social group) may contribute to member wellbeing, depression, meaning in life, and loneliness indirectly through the mechanisms of psychological safety and social network quality. Specifically, we found evidence that social network quality within one’s Shed may be a key mechanism through which identity leadership is associated with mental health outcomes, with psychological safety and social identity contributing through their capacity to also improve Shed social network quality. Our findings extend previous literature and have significant practical and theoretical implications for future research, such as exploring interventions to improve identity leadership and social network quality within Men’s Sheds.

## Supplemental Material

Supplemental Material - Leading by Example: Identity Leadership and Mental Health in Men’s Sheds Members Supplemental Material for Leading by Example: Identity Leadership and Mental Health in Men’s Sheds Members by Clarke, J. J.1,* ORCID, Haslam, S. A.2, Sharman, L. S.2, Guerrini, B.1, Holmes, K.1, Talbot, R.3, Wild, J.3, and McEvoy, P. M. in Journal of Applied Gerontology.

## References

[bibr1-07334648241289020] Alsubheen S. A. Oliveira A. Habash R. Goldstein R. Brooks D. (2023). Systematic review of psychometric properties and cross-cultural adaptation of the University of California and Los Angeles Loneliness Scale in adults. Current Psychology, 42(6), 1–15. 10.1007/s12144-021-02494-w 34785877 PMC8586628

[bibr2-07334648241289020] Bonnín C. M. Yatham L. N. Michalak E. E. Martínez-Arán A. Dhanoa T. Torres I. Santos-Pascual C. Valls E. Carvalho A. F. Sánchez-Moreno J. Valentí M. Grande I. Hidalgo-Mazzei D. Vieta E. Reinares M. (2018). Psychometric properties of the Well-Being Index (WHO-5) Spanish Version in a sample of euthymic patients with bipolar disorder. Journal of Affective Disorders, 228, 153–159. 10.1016/j.jad.2017.12.006 29248821

[bibr3-07334648241289020] Bui B. K. H. (2020). The relationship between social network characteristics and depressive symptoms among older adults in the United States: Differentiating between network structure and network function. Psychogeriatrics, 20(4), 458–468. 10.1111/psyg.12530 32045499

[bibr4-07334648241289020] Chui R. C. F. (2018). The role of meaning in life for the quality of life of community-dwelling Chinese elders with low socioeconomic status. Gerontology & Geriatric Medicine, 4, 233372141877414. 10.1177/2333721418774147 PMC5952284 29780856

[bibr5-07334648241289020] Clarke J. J. Talbot R. Holmes K. Wild J. Ashley J. McEvoy P. M. (2023). Social anxiety, behavioural activation, and depression risk in older men: Protection through Men’s Shed membership. Health Promotion International, 38(6), daad180. 10.1093/heapro/daad180 38150221

[bibr6-07334648241289020] Cruwys T. Haslam S. A. Dingle G. A. Haslam C. Jetten J. (2014). Depression and social identity: An integrative review. Personality and Social Psychology Review, 18(3), 215–238. 10.1177/1088868314523839 24727974

[bibr7-07334648241289020] Dingle G. A. Sharman S. L. (2022). Social prescribing: A review of the literature. In Menzies R. G. Menzies R. E. Dingle G. A. (Eds.), Existential concerns and cognitive-behavioral procedures: An integrative approach to mental health (pp. 135–149). Springer. 10.1007/978-3-031-06932-1_8

[bibr8-07334648241289020] Domènech-Abella J. Mundó J. Switsers L. van Tilburg T. Fernández D. Aznar-Lou I. (2021). Social network size, loneliness, physical functioning and depressive symptoms among older adults: Examining reciprocal associations in four waves of the Longitudinal Aging Study Amsterdam (LASA). International Journal of Geriatric Psychiatry, 36(10), 1541–1549. 10.1002/gps.5560 33908639

[bibr9-07334648241289020] Edmondson A. (1999). Psychological safety and learning behavior in work teams. Administrative Scient Quarterly, 44(2), 350–383. 10.2307/2666999

[bibr10-07334648241289020] Edmonson A. C. Lei Z. (2014). Psychological safety: The history, renaissance, and future of an interpersonal construct. Annual Review of Organizational Psychology and Organizational Behavior, 1(1), 23–43. 10.1146/annurev-orgpsych-031413-091305

[bibr11-07334648241289020] Flood P. Blair S. (2013). Men’s Sheds in Australia: Effects on physical health and mental well-being. Beyond Blue.

[bibr12-07334648241289020] Foettinger L. Albrecht B. M. Altgeld T. Gansefort D. Recke C. Stalling I. Bammann K. (2022). The role of community-based men’s Sheds in health promotion for older men: A mixed-methods systematic review. American Journal of Men’s Health, 16(2), 15579883221084490. 10.1177/15579883221084490 PMC8928410 35287514

[bibr13-07334648241289020] Ford S. Scholz B. Lu V. N. (2015). Social shedding: Identification and health of Men’s Sheds users. Health Psychology, 34(7), 775–778. 10.1037/hea0000171 25365412

[bibr14-07334648241289020] Fransen K. McEwan D. Sarkar M. (2020). The impact of identity leadership on team functioning and well-being in team sport: Is psychological safety the missing link? Psychology of Sport and Exercise, 51, Article 101763. 10.1016/j.psychsport.2020.101763

[bibr15-07334648241289020] Galdas P. Cheater F. Marshall P. (2005). Men and health help-seeking behaviour: Literature review. Journal of Advanced Nursing, 49(6), 616–623. 10.1111/j.1365-2648.2004.03331.x 15737222

[bibr16-07334648241289020] Gammage R. J. Foster J. L. (2017). Leadership in community mutual support groups for mental health: A qualitative case study from the leaders’ perspective. Journal of Community & Applied Social Psychology, 27(6), 463–475. 10.1002/casp.2327

[bibr17-07334648241289020] Gray J. Kim J. Ciesla J. R. Yao P. (2014). Rasch analysis of the lubben social network scale – 6 (LSNS-6). Journal of Applied Gerontology, 35(5), 508–528. 10.1177/0733464814560468 25428591

[bibr18-07334648241289020] Haslam C. Jetten J. Cruwys T. Dingle G. A. Haslam S. A. (2018). The new psychology of health: Unlocking the social cure. Routledge.

[bibr19-07334648241289020] Haslam S. A. Haslam C. Cruwys T. Jetten J. Bentley S. Fong P. Steffens N. K. (2022). Social identity makes group-based social connection possible: Implications for loneliness and mental health. Current Opinion in Psychology, 43, 161–165. 10.1016/j.copsyc.2021.07.013 34403958

[bibr20-07334648241289020] Haslam S. A. Reicher S. D. (2016). Rethinking the psychology of leadership: From personal identity to social identity. Dædalus, 145(3), 21–34. 10.1162/daed_a_00394

[bibr21-07334648241289020] Haslam S. A. Reicher S. D. Platow M. J. (2011). The new psychology of leadership: Identity, influence and power (1st ed.). Psychology Press.

[bibr22-07334648241289020] Haslam S. A. Reutas J. Bentley S. V. McMillan B. Lindfield M. Luong M. Peters K. Steffens N. K. (2023). Developing engaged and ‘teamful’ leaders: A randomized controlled trial of the 5R identity leadership program. PLoS One, 18(5), Article e0286263. 10.1371/journal.pone.0286263 37228145 PMC10212178

[bibr23-07334648241289020] Haslam S. A. Steffens N. K. Peters K. Boyce R. A. Mallett C. J. Fransen K. (2017). A social identity approach to leadership development: The 5R program. Journal of Personnel Psychology, 16(3), 113–124. 10.1027/1866-5888/a000176

[bibr24-07334648241289020] Kelly D. Steiner A. Mason H. Teasdale S. (2019). Men’s Sheds: A conceptual exploration of the causal pathways for health and well-being. Health and Social Care in the Community, 27(5), 1147–1157. 10.1111/hsc.12765 31206945 PMC6772158

[bibr25-07334648241289020] Kroenke K. Spitzer R. L. Williams J. B. W. (2001). The PHQ-9: Validity of a brief depression severity measure. Journal of General Internal Medicine, 16(9), 606–613. 10.1046/j.1525-1497.2001.016009606.x 11556941 PMC1495268

[bibr26-07334648241289020] Lubben J. Blozik E. Gillmann G. Iliffe S. von Renteln Kruse W. Beck J. C. Stuck A. E. (2006). Performance of an abbreviated version of the Lubben Social Network Scale among three European community-dwelling older adult populations. The Gerontologist, 46(4), 503–513. 10.1093/geront/46.4.503 16921004

[bibr27-07334648241289020] McEvoy P. M. Holmes K. Smith B. Bullen J. Chiu V. W. Wild J. Ashley J. Talbot R. (2023). Pathways from Men’s Shed engagement to wellbeing, health-related quality of life and lower loneliness. Health Promotion International, 38(4), daad084. 10.1093/heapro/daad084 37584668

[bibr28-07334648241289020] McGrath A. Murphy N. Egan T. Ormond G. Richardson N. (2022). Understanding shedders: Which socio-demographic, health and wellbeing characteristics best inform appropriate health promotion action in Men’s Sheds and a ‘Shed for Life’? Health Promotion Journal of Australia, 34(1), 156–168. 10.1002/hpja.649 36692862

[bibr29-07334648241289020] McGrath A. Murphy N. Richardson N. (2022b). ‘Sheds for life’: Delivering a gender-transformative approach to health promotion in Men’s Sheds. Health Promotion International, 37(6), 1–14. 10.1186/s12889-021-10823-8 36300699

[bibr30-07334648241289020] Mertens N. Boen F. Steffens N. K. Haslam S. A. Bruner M. Barker J. B. Slater M. J. Fransen K. (2021). Harnessing the power of ‘us’: A randomized wait-list controlled trial of the 5R shared leadership development program (5rs) in basketball teams. Psychology of Sport and Exercise, 54(1), Article 101936. 10.1016/j.psychsport.2021.101936

[bibr31-07334648241289020] Milligan C. Payne S. Bingley A. Cockshott Z. (2015). Place and wellbeing: Shedding light on activity interventions for older men. Ageing and Society, 35(1), 124–149. 10.1017/S0144686X13000494

[bibr32-07334648241289020] Montano D. Reeske A. Franke F. Hüffmeier J. (2017). Leadership, followers’ mental health and job performance in organizations: A comprehensive meta-analysis from an occupational health perspective. Journal of Organizational Behavior, 38(3), 327–350. 10.1002/job.2124

[bibr33-07334648241289020] Montano D. Schleu J. E. Hüffmeier J. (2023). A meta-analysis of the relative contribution of leadership styles to followers’ mental health. Journal of Leadership & Organizational Studies, 30(1), 90–107. 10.1177/15480518221114854

[bibr34-07334648241289020] Newman A. Donohue R. Eva N. (2017). Psychological safety: A systematic review of the literature. Human Resource Management Review, 27(3), 521–535. 10.1016/j.hrmr.2017.01.001

[bibr35-07334648241289020] Nurmi M. A. Mackenzie C. S. Roger K. Reynolds K. Urquhart J. (2018). Older men’s perceptions of the need for and access to male-focused community programmes such as Men’s Sheds. Ageing and Society, 38(4), 794–816. 10.1017/S0144686X16001331 29551844 PMC5848757

[bibr36-07334648241289020] O’Donovan R. McAuliffe E. (2020). A systematic review of factors that enable psychological safety in healthcare teams. International Journal for Quality in Health Care, 32(4), 240–250. 10.1093/intqhc/mzaa025 32232323

[bibr37-07334648241289020] Postmes T. Haslam S. A. Jans L. (2013). A single-item measure of social identification: Reliability, validity and utility. British Journal of Social Psychology, 52(4), 597–617. 10.1111/bjso.12006 23121468

[bibr38-07334648241289020] Rose L. M. Zask A. Burton L. J. (2017). Psychometric properties of the meaning in life questionnaire (MLQ) in a sample of Australian adolescents. International Journal of Adolescence and Youth, 22(1), 68–77. 10.1080/02673843.2015.1124791

[bibr39-07334648241289020] Russell D. Peplau L. A. Cutrona C. E. (1980). The revised UCLA loneliness scale: Concurrent and discriminant validity evidence. Journal of Personality and Social Psychology, 39(3), 472–480. 10.1037//0022-3514.39.3.472 7431205

[bibr40-07334648241289020] Steffens N. K. Haslam S. A. Reicher S. D. Platow M. J. Fransen K. Yang J. Ryan M. K. Jetten J. Peters K. Boen F. (2014). Leadership as social identity management: Introducing the Identity Leadership Inventory (ILI) to assess and validate a four-dimensional model. The Leadership Quarterly, 25(5), 1001–1024. 10.1016/j.leaqua.2014.05.002

[bibr41-07334648241289020] Steger M. F. Franzier P. Oishi S. Kaler M. (2006). The meaning in life questionnaire: Assessing the presence of and search for meaning in life. Journal of Counselling Psychology, 53(1), 80–93. 10.1037/0022-0167.53.1.80

[bibr42-07334648241289020] Suragarn U. Hain D. Pfaff G. (2021). Approaches to enhance social connection in older adults: An integrative review of literature. Aging and Health Research, 1(3), Article 100029. 10.1016/j.ahr.2021.100029

[bibr43-07334648241289020] Titov N. Dear B. F. McMillan D. Anderson T. Zou J. Sunderland M. (2011). Psychometric comparison of the PHQ-9 and BDI-II for measuring response during treatment of depression. Cognitive Behavior Therapy, 40(2), 126–136. 10.1080/16506073.2010.550059 25155813

[bibr44-07334648241289020] World Health Organization . (1998). Wellbeing measures in primary healthcare/the depcare project: Reported on a WHO meeting. https://www.euro.who.int/__data/assets/pdf_file/0016/130750/E60246.pdf

