# Supplementary material for: Leading by Example: Identity Leadership and Mental Health in Men’s Sheds Members
Source: J Appl Gerontol. 2024 Sep 30;44(5):815–24. doi: 10.1177/07334648241289020 (PMC11997286; doi:10.1177/07334648241289020)
Supplement: Supplemental Material - Leading by Example: Identity Leadership and Mental Health in Men’s Sheds Members [file sj-pdf-1-jag-10.1177_07334648241289020.pdf]

Correlation Matrix

	IL	SO	PS	SSNQ	Wellbeing	Dep	MIL
IL	-						
SI	.52**	-					
PS	.50**	.30**	-				
SSNQ	.40**	.59**	.41**	-			
Wellbeing	.23*	.27**	.27**	.41**	-		
Dep	-.114	-.15	-.23*	-.35**	-.69**	-	
MIL	.23*	.33**	.27**	.42**	.47**	-.40**	-
Loneliness	-.34**	-.32**	-.36**	-.45**	-.43**	.41**	-.46**

Note. IL = Identity Leadership, SI = Social Identity, PS = Psychological Safety, SSNQ = Shed Social Network Quality, Dep = Depression, MIL = Meaning in Life, * $p < .01$, ** $p < .001$.
